# Indocyanine Green for Leakage Control in Isolated Limb Perfusion

**DOI:** 10.3390/jpm11111152

**Published:** 2021-11-05

**Authors:** Isabel Zucal, Sebastian Geis, Lukas Prantl, Silke Haerteis, Thiha Aung

**Affiliations:** 1 Centre of Plastic, Aesthetic, Hand and Reconstructive Surgery, University Clinic of Regensburg, 93053 Regensburg, Germany; isabel.zucal@ksa.ch (I.Z.); sebastian.geis@ukr.de (S.G.); lukas.prantl@ukr.de (L.P.); 2 Institute for Molecular and Cellular Anatomy, University of Regensburg, 93053 Regensburg, Germany; silke.haerteis@ur.de; 3 Faculty of Applied Healthcare Science, Deggendorf Institute of Technology, 94469 Deggendorf, Germany

**Keywords:** cytostatic agents, doxorubicin, indocyanine green, oncology, perfusion, surgical oncology

## Abstract

Sarcomas are characterized by a high metastatic potential and aggressive growth. Despite surgery, chemotherapy plays an important role in the treatment of these tumors. Optimal anti-cancer therapy with maximized local efficacy and minimized systemic side effects has been the object of many studies for a long time. To improve the local efficacy of anti-tumor therapy, isolated limb perfusion with high-dose cytostatic agents has been introduced in surgical oncology. In order to control the local distribution of substances, radiolabeled cytostatic drugs or perfusion solutions have been applied but often require the presence of specialized personnel and result in a certain exposure to radiation. In this study, we present a novel strategy using indocyanine green to track tumor perfusion with high-dose cytostatic therapy. In a rat cadaver model, the femoral vessels were cannulated and connected to a peristaltic pump to provide circulation within the selected limb. The perfusion solution contained indocyanine green and high-dose doxorubicin. An infrared camera enabled the visualization of indocyanine green during limb perfusion, and subsequent leakage control was successfully performed. Histologic analysis of sections derived proximally from the injection site excluded systemic drug dispersion. In this study, the application of indocyanine green was proven to be a safe and cost- and time-efficient method for precise leakage control in isolated limb perfusion with a high-dose cytostatic agent.

## 1. Introduction

Sarcomas are tumors that are characterized by an aggressive growth pattern, early metastasis, and poor prognosis [1,2,3]. Oncologic surgery comprises many medical disciplines and therapeutic approaches. Surgical resection is an important aspect in cancer treatment and often involves additional lymphadenectomy of sentinel lymph nodes and downstream lymph node stations [4]. In some cases, radiotherapy is performed for local disease control and to increase functional outcomes [5]. However, despite lymph node resection, radiation increases the risk of lymphatic vessel damage and lymphatic complications [6,7]. Moreover, an exclusively surgical approach often results in the loss of the entire limb. In the last few decades, neoadjuvant chemotherapy and adjuvant chemotherapy have been added to the treatment protocols of sarcomas. A combination of doxorubicin, cisplatin, and methotrexate with leucovorin and ifosfamide represents the most commonly used combination of chemotherapeutic agents for the treatment of sarcomas [8]. However, systemic drug application is accompanied by systemic toxicity.

In the past few years, there has been a great effort within oncologic research to develop improved drug delivery systems and application modes that enable a high local efficacy while keeping systemic toxic effects at the lowest level possible. In this regard, isolated limb perfusion (ILP) has been described as a technique that permits the local application of high-dose cytostatic drugs into tumors or metastases located within extremities [9,10,11]. The technique is mostly used for the treatment of locally advanced melanoma and unresectable primary or recurrent sarcomas of the limb [12,13,14]. Depending on the affected limb, the perforating vessels are cannulated and connected to an extracorporeal device, which provides circulation to the affected limb with high doses of cytostatic agents. The vessels can be cannulated at different levels: iliac, iliofemoral, femoropopliteal, and popliteal [15]. Additionally, local hyperthermia is achieved by placing a thermal blanket around the affected extremity to increase the cytotoxic effects [12,15].

Radionuclide-labeled cytostatic drugs or perfusion solutions have been used for adequate leakage control at the cannulation site and for the control of the correct application of such procedures [16,17]. After the correct cannulation is confirmed, high-dose cytostatic drugs, such as TNFα + melphalan [9,18] are added to the solution, which is subsequently injected into the chosen region. However, this results in a certain amount of exposure to radiation [19,20], high costs, and the requirement of specialized personnel to carry out this procedure.

Doxorubicin, an anthracycline antibiotic, represents a substantial pillar in treatment protocols for solid tumors such as sarcomas [21]. However, cardiotoxicity and nephrotoxicity are some of the most common systemic side effects [21]. As these solid tumors usually affect the extremities, a limb salvage procedure is highly desirable. ILP presents one possibility to increase operability and to preserve functionality. Isolated limb infusion with doxorubicin has been described for the treatment of soft tissue sarcomas with promising results in this regard [22]. Due to the fluorescent property of doxorubicin, it can be visualized and traced by fluorescence microscopy, making it very suitable for preclinical and clinical studies. Here, we propose a simple, safe, and cost- and time-efficient technique for leakage control in ILP with high-dose doxorubicin.

## 2. Materials and Methods

Rat cadaver models were placed on a corkboard in a supine position underneath a microscope; the skin was incised longitudinally on the medial part of the thigh. The femoral vessels were carefully prepared, dissected, and cannulated. Cannulas were kept in place with clamps and were attached to a 12,000 Varioperpex peristaltic pump (LKB Bromma, Sweden) that provided circulation within the cannulated extremity. The maximum perfusion speed was 5 mL/min. A plastic loop was placed around the groin and served as a tourniquet. The femoral artery was utilized for inflow and the femoral vein for outflow of the perfusion fluid. The dissection and cannulation of the vessels were performed under a Leica M205A stereomicroscope, which conveys a maximal resolution of 1050 lp/mm and a zoom range of 7.8×–160×. Two different beam path lengths provide a high level of depth of focus, whereby orientation within a multidimensional space is enabled. For the microsurgical dissection and cannulation of the femoral vessels, customized instruments from Redam instruments GmbH were utilized. The rat cadavers we used were Munich–Wistar–Froemter rats, aged between 7 and 10 weeks, previously used for other experiments. The procedures were carried out within 1–4 h after the death of the rats. If the experiment could not be carried out immediately after death, the rats were placed in the freezer at −20 °C and thawed in a water bath for 1 h at 37 °C.

The peristaltic pump perfused the circulatory system of the cannulated limb with approximately 50 mL of perfusion solution containing 47 mL of 1× PBS (Sigma Aldrich, St. Louis, MO, USA), 1000 IU of heparin with a concentration of 5000 IU/mL (B. Braun Melsungen AG, Melsungen, Germany), 2 mL of indocyanine green (ICG) with a concentration of 2.5 mg/mL (Diagnostic Green GmbH, Aschheim, Germany), and high-dose doxorubicin (2 mg/mL). The Elevision IR (VSIII) fluorescence system (Medtronic, Minneapolis, MN, USA) was used for the fluorescence imaging in perfusion recognition modes to visualize and trace the injected ICG. The same camera conditions were used for all acquisitions: exposure time was 40 Hz (=2.5 ms), and gain was 1. Fluorescence level was dependent on penetration depth and camera focus. ICG served as an indicator of leakage and confirmed the correct drug application. To provide a comparison between ILP and whole-body perfusion, the femoral vessels were also cannulated in the inverted direction, and after perfusion with the same solution, fluorescent imaging was performed. Perfusion time was between 15 and 20 min. The experiment was repeated five times.

### Fluorescence Microscopy

For further proof of correct drug application, tissue samples (thigh muscle) were taken from anatomical regions proximal to the cannulation site and from the contralateral limb to exclude leakage and systemic dispersion. As a positive control, three samples were obtained from the perfused limb. These samples were immerged in 4% PFA in PBS (pH 7.4) for 16 days, then washed in PBS three times for 30 min, and immersed in EDTA decalcification solution for 90 min. Then, samples were embedded in Neg-50^TM^ frozen section medium (Thermo Fisher Scientific, Waltham, MA, USA). Finally, samples were cryosectioned and analyzed by fluorescence microscopy with Axiovert 200 M (Zeiss, Oberkochen, Germany). Microscopic analysis enabled the visualization of doxorubicin via fluorescence after excitation at 560 nm (100 ms, mCherry mode) with DAPI counterstain (20 ms, DAPI mode) at 40× magnification. Magnification was further increased to 200× utilizing ImageJ (LOCI, University of Wisconsin, Madison, WI, USA).

## 3. Results

**Figure 1:**
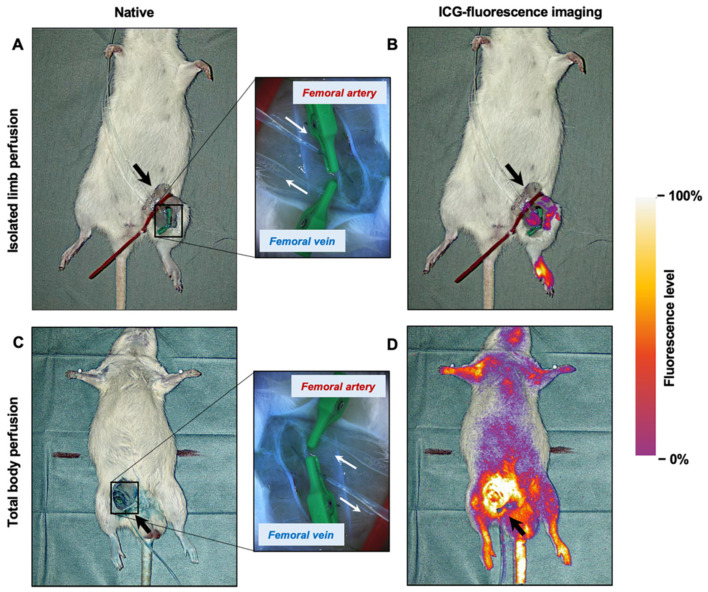
ICG-fluorescence imaging as indicator of ILP. (**A**) The native image shows the cannulation site: the femoral artery and vein are cannulated for perfusion inflow and outflow. The arrow indicates the direction of the flow. Clamps are used to keep the cannulas in place, and a magnification (4×) of the cannulation site is provided for a better overview. (**B**) ILP is demonstrated with ICG fluorescence imaging. (**C**) For comparison, a native picture of a rat cadaver with whole-body perfusion is shown. In this case, the direction of flow is inverted (arrow). A magnification of the cannulation site is provided (4×). (**D**) ICG fluorescence imaging of whole-body perfusion evokes a glowing signal of the entire body, while the cannulated extremity is spared. Fluorescence level is indicated by brightness and color: areas with the smallest penetration depth result in the highest fluorescence signal, e.g., areas uncovered by fur or skin, such as the cannulation site.

The femoral vessels were successfully dissected and cannulated in all rats. Each limb was perfused after connecting the cannulas to the peristaltic pump. ICG-fluorescence imaging showed locally restricted distribution of the perfusion fluid containing the cytostatic drug by causing the cannulated limb to emit a glowing signal (Figure 1A,B). No leakage was observed. In contrast to ILP, ICG-fluorescence imaging of whole-body perfusion after inverting the cannulation direction of the femoral vessels showed a glowing signal over the entire body (Figure 1C,D). In areas with less or no fur or skin, such as the paws, the belly, the tail, or the cannulation site, a higher fluorescence level was recorded. Fur contamination with perfusion fluid around the cannulation site also resulted in an increased fluorescence level.

**Figure 2:**
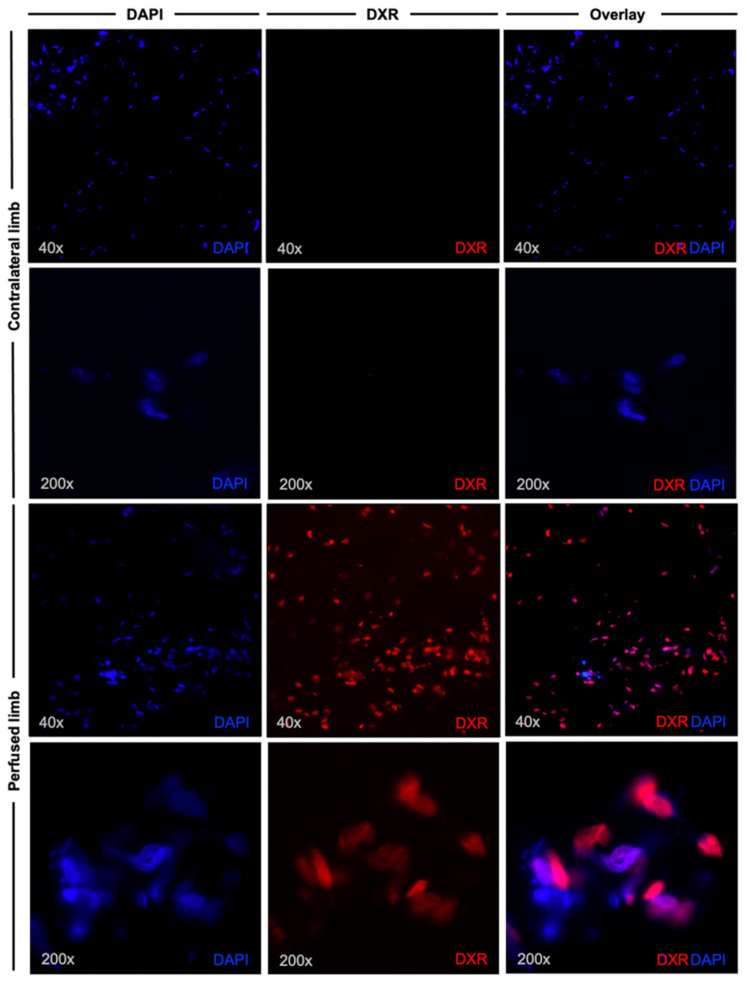
Fluorescence microscopy. In the first and second row, images of the sample derived from the contralateral limb are shown at 40× and 200× magnification, respectively. No intracellular doxorubicin (DXR) accumulation was observed, and nuclei were counterstained with DAPI. In the third and fourth row, fluorescence images of a sample from the perfused limb are displayed. Microscopic analysis displays intracellular doxorubicin accumulation.

Fluorescence microscopy of samples derived proximally from the injection site of the contralateral limb showed no intracellular doxorubicin accumulation (Figure 2, first two rows), giving proof of no systemic drug dispersion. On the other hand, microscopic analysis of the samples obtained from the perfused limb showed intracellular doxorubicin accumulation (Figure 2, last two rows).

## 4. Discussion

Sarcomas are aggressive tumors that often affect the extremities with high risk of metastatic spread. Among the different types of sarcomas, one differentiates between soft tissue sarcoma; bone tumors, such as osteosarcoma, chondrosarcoma, or Ewing-sarcoma; and gastrointestinal stromal tumors. Sarcomas are characterized by high inter- and intra-tumoral diversity at a genetic level, rendering treatment very difficult. Because of their aggressiveness and the potential for early metastasis, a safe tumor resection and lymphadenectomy of affected lymph nodes in combination with neoadjuvant and adjuvant chemotherapy are crucial. However, this radical surgical removal results in a high rate of lymphatic complications despite the risk of limb loss. With regard to complications related to the lymphatic system, an incidence of lymphedema of about 29% has been found following limb salvage of soft tissue sarcoma located within extremities [23]. The number of resected lymph nodes [24,25,26], tumor localization within the thigh, tumor depth, and size > 5 cm were identified as risk factors for the development of lymphedema [23,27]. Moreover, for local disease control, patients frequently undergo radiotherapy [28], and, as mentioned before, this also contributes to the development of lymphatic complications. To treat lymphatic complications, surgery such as lymphovenous anastomosis is often required [29]. Furthermore, limb preservation remains highly desirable, and complex microsurgical functional reconstructions have been developed for this purpose, such as the Borggreve rotationplasty, which provides a reconstruction of the knee joint after resection of osteosarcoma of the distal femur [30,31], or the Winkelmann procedure, which consists of a clavicula-based reconstruction of the humerus [32].

In Third World countries, such complex treatments and associated costs are neither available nor sustainable. For instance, amputation is still the most common therapy of osteosarcoma in Third World countries, whereas limb salvage procedures in combination with adjuvant and neoadjuvant chemotherapy became the momentary standard therapy in surgical oncology [33]. Poor countries in particular may profit from cost-, time-, and personnel-efficient techniques of cytostatic drug application, such as ILP.

Cancer therapy often requires the application of high-dose cytostatic drugs in order to cause antitumoral effects, but, on the other hand, this also carries the risk of serious side effects. Thus, in the past few decades, there has been an increased effort to develop therapies that avoid these side effects, such as the administration of corticosteroids and colony stimulating factors [34,35]. ILP has been developed to minimize systemic effects of cytostatic agents and maximize the local drug concentration [9,10,11]. Additional hyperthermia is thought to enhance the uptake of cytostatic agents, thereby increasing the effects on the tumor [12]. However, for safe application of these highly concentrated cytostatic agents, leakage control is of utmost importance. In this regard, the addition of radionuclides to the perfusion solution has already been described as a method for sufficient leakage control [16,17], but this causes a certain amount of radiation and requires specialized personnel [19,20]. Although doxorubicin has been used for decades, it was recently replaced by TNFα + melphalan, which is now the standard therapy in ILP for sarcomas. In fact, higher toxicities and less activity are attributed to the application of doxorubicin compared to TNFα + melphalan [36]. In our experiments, we used doxorubicin because of its fluorescent property to track drug dispersion after ILP. Cytostatic drug toxicity in ILP is related to the amount of leakage into systemic circulation, and, in addition to local leakage control, high intravenous hydration promotes the washout effect of these drugs, reducing systemic toxicity. Local complications affecting the perfused limb include deep venous thrombosis [37], limb edema, and erythema, deep tissue damage with functional impairment and compartment syndrome [38]. In our experiments, we observed limb edema in all rats. No blisters or epidermolysis were present. However, as we performed the experiment on rat cadavers, complications such as functional impairment or compartment syndrome could not be assessed.

The innovative aspect of this experiment is the utilization of ICG for leakage control in isolated limb perfusion. In a rat cadaver model, the femoral vessels were cannulated and attached to a peristaltic pump for the establishment of circulation within the cannulated extremity. The perfusion fluid contained PBS, 1000 IE of heparin, 2 mL of ICG, and high doses of doxorubicin. An infrared camera was used to visualize ICG, which served as an indicator of leakage and depicted the localized drug application in ILP. Histologic analysis of anatomical regions proximal to the cannulated limb provided proof of the correct application of the drug by indicating that there was no systemic dispersion. Another innovative aspect of this experiment is the usage of rat cadaver models. Fluid-resuscitated rat cadavers represent an animal-friendly research model with regard to the 3R principles of replacement, refinement, and reduction of animal models in research and medical training. Therefore, this model could be used for further infusion/perfusion studies in the future.

ICG has a broad application spectrum in surgery. It is an amphiphilic fluorescence dye, which is used for the purpose of intravenous injection into patients. Once in the circulatory system, 98% of the injected ICG binds to plasma protein, and 2% remains free in the blood serum until it is metabolized by the liver and subsequently excreted into bile [39]. The dye has a half-life of generally 3–4 min that depends on the vascularity of the investigated organ [39]. Because of the rapid clearance rate, the dye can be used for several injections during one procedure [39]. In addition, ICG is a well-established substance in clinics and surgery and is also characterized by a low side effect profile, ensuring the safety of its application [40,41]. Moreover, it is easily available and applicable to the perfusion solution used for ILP. For instance, it is well established in supermicrosurgery, as it permits the visualization of lymphatics, and in reconstructive surgery where it is used to assess the perforators, tissue perfusion, and the viability of microvascular anastomoses [41,42,43]. In oncologic surgery, it is used for sentinel lymph node mapping and intraoperative identification of tumors [44]. Due to intratumoral injection, exact tumor margins can be visualized, ensuring safe resection without damage of surrounding tissue [45,46]. Moreover, it can be injected into the epineurium of nerves to improve the intraoperative visualization and to avoid accidental damage [47]. An important limitation of the use of ICG, however, is the depth of detection. In fact, structures deeper than 5–8 mm cannot be visualized [48]. Nevertheless, the detection depth is sufficient for leakage control of the cannulation site.

This study has some limitations. Although no systemic cytostatic drug dispersion was found in our experiments after approximately 15 min of infusion, systemic dispersion will likely occur due to the vascular collaterals if no tourniquet is placed at the level of the groin or the axillary fossa. Another limitation of this model is post-mortem clot formation and incomplete limb and/or whole-body perfusion. Although our perfusion fluid contained heparin, complete thrombus dissolution was not always possible, especially in thawed rat cadavers. We observed that the longer we waited after the rats had deceased, the higher the probability of clot formation. Freezing rat cadavers allowed for preservation for weeks or months, but the probability of vascular obstruction was higher compared to unfrozen and rapidly perfused rats. According to Okazaki et al., heparinization was described post-mortem in transplanted canine lungs for the prevention of microthrombi [49]. In this study, the optimal post-mortem heparinization time was 30 min after cardiac arrest [49]. Moreover, Marchioro et al. described the use of heparin in extracorporeal perfusion for the obtainment of post-mortem homografts in animal models [50].

As an alternative to the more invasive ILP, requiring open dissection of the vessels and the use of large-caliber catheters, isolated limb infusion has been suggested as a valid, minimally invasive, and safe alternative. In addition, when performing isolated limb infusion, small-caliber catheters are placed percutaneously [12,13]. Moreover, perfusion fluid is not oxygenated in isolated limb infusion, providing a hypoxic milieu, which enhances cytotoxic drug effects [12]. In our experiment, a percutaneous procedure could not be performed due to the small diameter of the femoral vessels of rats (approximately 0.54–0.56 mm [51]).

## 5. Conclusions

The use of ICG for leakage control in ILP for cancer treatment enables a safe application of high-dose cytostatic drugs by avoiding the application of radionuclides for this purpose. This makes it a more cost-, time-, and personnel-efficient technique, and no cooperation with nuclear medicine specialists is required.

## Data Availability

The data presented in this study are available on request from the corresponding author.
